# Reduced-Cost Genotyping by Resequencing in Peanut Breeding Programs Using Tecan Allegro Targeted Resequencing V2

**DOI:** 10.3390/genes15111364

**Published:** 2024-10-24

**Authors:** Cheng-Jung Sung, Roshan Kulkarni, Andrew Hillhouse, Charles E. Simpson, John Cason, Mark D. Burow

**Affiliations:** 1 Department of Plant and Soil Science, Texas Tech University, Lubbock, TX 79409, USA; cjsung0130@gmail.com; 2 Department of Agronomy, Iowa State University, Ames, IA 50011, USA; roshank@iastate.edu; 3 Texas A&M Institute for Genome Sciences and Society, College Station, TX 77843, USA; hillhouse@tamu.edu; 4 Department of Soil and Crop Sciences, Texas A&M AgriLife Research, Stephenville, TX 76401, USA; charles.simpson@ag.tamu.edu (C.E.S.); john.cason@ag.tamu.edu (J.C.); 5 Department of Soil and Crop Sciences, Texas A&M AgriLife Research, Lubbock, TX 79403, USA

**Keywords:** genotyping, resequencing, SNP, target, genome

## Abstract

The identification of informative molecular markers is useful for linkage mapping and can benefit genome-wide association studies by providing fine-scale information about sequence variations. However, high-throughput genotyping approaches are not cost-effective for labs that require frequent use, such as breeding programs that need to perform genotyping on large populations with hundreds of individuals. The number of single nucleotide polymorphism markers generated by those approaches can be far more than needed for most breeding programs; instead, breeders focus on the use of at most hundreds of polymorphic molecular markers for analysis. To help make use of molecular markers a routine tool for breeding programs, we aim to develop a cost-effective genotyping system by using the Tecan Allegro Targeted Resequencing V2 kit. This provides a customized probe design, which indicates that all the DNA fragments synthesized are known targets. SNPs obtained from previous peanut next-generation sequencing data were pre-filtered and selected as targets. These SNP targets were polymorphic among different tetraploid accessions and were selected to be distinguishable from paralogs. A total of 5154 probes were designed to detect 2770 SNP targets and were tested on 48 accessions, which include some closely related sister lines from a breeding population. The results indicated that genotyping by a targeted resequencing approach reduced the cost from around USD 28 (SNP chip and GBS) to USD 18 per sample, while providing polymorphic markers with accurate SNP calls. With this cost-effective genotyping platform, pre-selected SNP markers can be used effectively and routinely for more breeding programs.

## 1. Introduction

Genotyping has been used broadly for years in multiple scientific programs to provide deoxyribonucleic acid (DNA) information by detecting sequence variations. These genetic variations can be designed into molecular markers and are useful for research such as linkage mapping, quantitative trait locus (QTL) analysis, genome-wide association studies (GWAS), and marker-assisted selection (MAS). Traditional methods of genotyping use molecular markers by running gel electrophoresis, which is now considered low-throughput due to the limited number of samples per run and the need for a larger amount of DNA, extra time, and effort [1]. High-throughput genotyping approaches, including whole genome sequencing (WGS) and single nucleotide polymorphism (SNP) chip DNA microarrays, usually generate tens of thousands or more markers. However, the number of SNPs generated from these approaches is far more than needed for most breeding programs; instead, breeders generally focus on the use of a few to hundreds of polymorphic molecular markers at most for linkage mapping and QTL analysis [2]. One limiting factor is that the statistical power is decided more by the population size than the number of markers [3]. Additionally, it is expensive for labs that require marker use as a routine tool, such as breeding programs that need to perform genotyping on large populations with hundreds of individuals. When the breeding population is large, the cost of genotyping could become too high. Therefore, developing a more economical genotyping system of marker analysis in breeding populations can help make the use of molecular markers a routine and affordable tool for breeding programs.

Peanut, *Arachis hypogaea*, as one of the most important legume crops, involves intensive breeding programs that require large populations with hundreds of accessions. The peanut genome is complex due to being polyploid, and the A and B genomes share around 98% similarity [4,5]. Before the Tifrunner reference genome was sequenced, employing Hi-C sequencing and Pac-Bio reads, the highly similar homoeologous and paralogous sequences increased the error rate of sequence alignment, which made accurate genotyping a challenging task [6]. When diploid sequences were the only available peanut genome references, SNP data were either analyzed by aligning sequence reads against diploid references or generated using the SNP chip array, which was also designed based on diploid references [6,7]. SNPs identified from the SNP chip array were designed to be only true SNPs, which were polymorphic among individuals at each pair of chromosomes, instead of between subgenomes [8]. However, version 1 of the SNP chip suffered from a lower-than-expected resolution of homoeologous sequences [9]. Version 2 used improved tools to enhance the resolution of such sequences [8].

Identifying true SNPs is important for accurate genotyping and mapping results. Since the tetraploid genome reference was released in 2017, it has provided more useful sequence information for projects that use tetraploid cultivated peanuts as materials [4]. Performing a basic local alignment search tool (BLAST) search for sequences against the Tifrunner genome reference can help identify highly similar sequences such as homoeologs and paralogs [6,10,11]. It is beneficial to distinguish sequences among homologous regions using a BLAST search for a more accurate sequence alignment and genotyping. To facilitate peanut breeding programs, it becomes important to develop an economical platform featuring flexibility to select the true SNP targets. Based on pre-existing SNP knowledge such as the polymorphism information content (PIC), number of paralogous regions, and significance associated with traits of interest, sequencing efforts would be made to cover genome regions that provide useful genetic information. This reduces the need to sequence for more reads and, therefore, reduces the cost of genotyping.

The Tecan Allegro Targeted Resequencing V2 kit (Tecan Trading AG, Männedorf, Switzerland) provides a customized probe design, which allows customers to select their own SNP targets and send target chromosome/position and reference genome sequence information to Tecan Allegro for designing the probes. In use, probes are ligated to the regions of the target sites, and the DNA fragments are amplified using reagents in the Tecan Allegro Targeted Resequencing V2 kit. The kit is compatible with Illumina sequencing platforms, such as HiSeq, MiSeq, MiniSeq, NextSeq, and NovaSeq, and has been used for at least 85 different species, such as mouse, sheep, horse, black poplar, and maize [12,13,14,15]. While the SNP chip and genotyping by sequencing (GBS) approaches can generate around 15 to 50 or more thousand SNPs, the numbers of markers are far more than a breeding program would need, and the cost is from USD 28 to USD 50 per sample. Using this genotyping by targeted resequencing system reduces the cost, with hundreds or thousands of useful SNP markers generated, making it a suitable and cost-effective genotyping platform for breeding programs. In addition, unlike a SNP chip, for which targets are fixed, or unlike restriction site-associated DNA sequencing (RAD-Seq), where targets cannot be selected, this targeted resequencing system provides the availability to customize probes.

This research proposes to develop a reduced-cost genotyping system by using the Tecan Allegro Targeted Resequencing V2 kit with a goal to reduce the cost to around USD 15 to USD 20 per sample. We aim to select true SNPs that are highly polymorphic as this is an important step for developing useful SNP markers, and this helps increase the accuracy rate of calling SNPs, with a reduced chance of misalignment due to short reads. In this research, we chose to select true SNPs that are distinguishable from their homoeologs and paralogs, using SNPs obtained from previous peanut next-generation sequencing (NGS) data, including the Arachis Affymetrix Axiom_Array2 SNP chip [8,9], RNA-Seq, Kompetitive Allele-Specific PCR (KASP) markers, and WGS data. To test the probes on multiple peanut accessions, we screened a subset of 48 peanut accessions, including check varieties, minicore accessions, closely related breeding lines, and some diploids. We expect these DNA samples would predict the utility of these markers among accessions of a large germplasm array. The identified SNP markers that are polymorphic among closely related peanut accessions and breeding populations will be useful for future peanut breeding programs.

## 2. Materials and Methods

### 2.1. Target Selection

**Figure 1:**
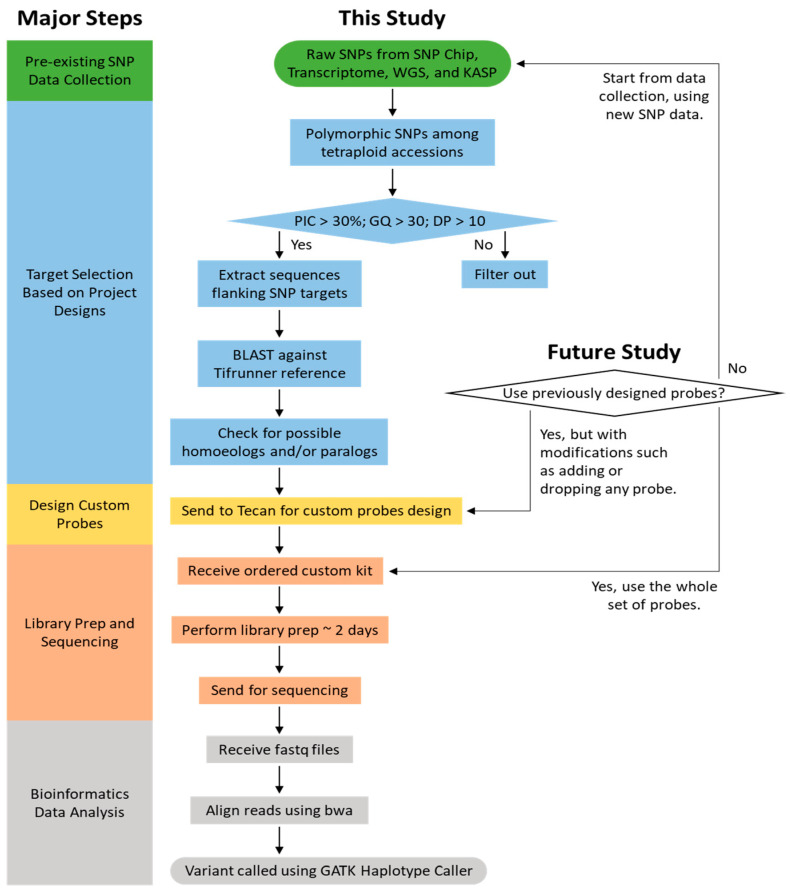
A flowchart to visually represent the research workflow.

The Tecan Allegro Targeted Resequencing V2 kit supports up to 2500 targets for 192 samples. To use it as a trial experiment (see Figure 1 for the workflow), the targets were selected from different sources including SNP chip targets, transcriptome sequence data, KASP markers, and WGS data.

#### 2.1.1. SNP Chip Data

The SNP chip data were collected from minicore samples of a drought experiment in 2017 (Sung and Burow, unpublished) using the Affymetrix Arachis Axiom_Array2 (Thermo Fisher Scientific Inc., Waltham, MA, USA) [8,9]. The information about the chromosomes and positions of the 8189 “PolyHighResolution” SNPs was first used to extract sequences flanking the two sides of each SNP site from the diploid reference sequences. Each extracted sequence was 601 bases long, with 300 bases flanking each side of the SNP site. These 8189 sequences were then aligned against Tifrunner reference (ver. 1) [4] using BLAST (ver. 2.11.0) [16,17,18] with an e-value of 1 × 10^−50^ to examine the number of matches. BLAST was performed locally on a Supermicro AMD server running the CentOS 6 operating system.

**Figure 2:**
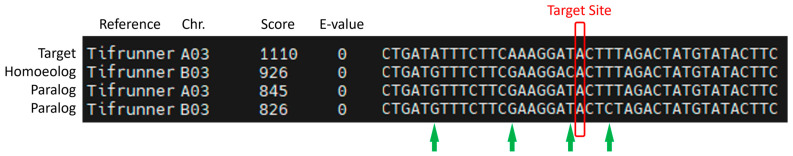
An example of target selection using BLAST.

Sequences with many BLAST matches may have many paralogs, which would result in greater difficulty in obtaining accurate sequence alignment and genotyping. To eliminate sequences with a large number of paralogs, we first dropped sequences that had more than 55 BLAST hits; this threshold removed approximately 40% of the targets. After removing sequences that had more than 55 BLAST hits against Tifrunner per locus, there were 5653 sequences left (Table 1). The SNP chip genotype data of these 5653 sequences were then used to calculate the polymorphism information content (PIC) value for each marker, using the genotypes of the minicore accessions to measure the informativeness of polymorphic DNA markers using the following equation [19,20,21]:

$$PIC=1-\sum_{i=1}^{n}p_{i^{2}}-2\sum_{i=1}^{n-1}\sum_{j=i+1}^{n}p_{i^{2}}p_{j^{2}}$$

where p_i_ was the frequency of the marker allele, and n was the number of different alleles. There were 3339 SNPs with PIC values greater than 30% (Table 1). Each of the 3339 sequences was then compared with its output from the BLAST results to filter out sequences that were identical to any homoeologous or paralogous region within the range of 300 bases on each side. An example of this step was shown in Figure 2 using BLAST results with four matches, where the first match with the highest score was the best match, and it was taken as the target sequence. For example, the target sequence was located on chromosome A03; therefore, one of the other matches on B03 could be the homoeologous sequence, and the rest would be paralogous sequences (Figure 2). It would be difficult to distinguish the target genotype from the homoeologs and paralogs if their sequences flanking the target site were identical. Therefore, it was important to make sure that none of the homoeologous/paralogous sequences was identical to the target sequence. Only targets with distinguishable homoeologs and paralogs were selected. In the example in Figure 2, the 21^st^ nucleotide (circled in red) was the target site, and the nucleotides that were pointed out by green arrows were the flanking SNPs, which made them distinguishable from the target sequence. The flanking SNPs were important, because they provided information to distinguish targets from homoeologs and paralogs and helped to select true SNPs.

After filtering out sequences that were identical to any homoeologous or paralogous region, each of the remaining 3178 sequences was then examined at the target site for the bases of the target and its possible homoeolog (Table 1). Due to the high similarity of A and B genomes, sequence reads that had different bases appearing at the target sites on both the target sequence and the homoeologous sequence could be aligned together in error and called as a SNP that was not a true SNP. To reduce the chance of selecting targets that are not true SNPs, we examined each of the 3178 sequences by comparing its target sites at the target sequence and the homoeologous sequence based on the Tifrunner reference output from BLAST results (see Figure 2 as an example). If different nucleotides are observed, there is a higher chance that the polymorphisms previously detected in the SNP chip analysis were just differences between targets and their homoeologous sites. After removing 1050 sequences that had polymorphisms between targets and their homoeologous sites, a total of 2128 sequences that had the same nucleotide at target sites were kept. These 2128 target sites were then selected for designing probes and were named with a beginning of “M_”.

#### 2.1.2. RNA-Seq Data

A total of 306,820 SNPs were identified from a de novo assembly of RNA-Seq data using the OLin transcriptome sequence as a reference [22]. Among the 12 tetraploid cultivated accessions (BSS56, COC155A (PI502111), COC224 (PI290538), COC367 (PI268868), COC559 (PI158854), PI648241, PI648242, Jupiter, New Mexico Valencia C (NMValC), OLin, Tamrun OL07, and UF439), there were 87,782 SNPs showing polymorphism, and 73,872 of them had PIC values greater than 30% (Table 2). Among them, there were 43,946 SNPs with a read depth (DP; summed across the 12 accessions) greater than 99 (Table 2). After removing SNPs with a DP of less than 10 for any accession, there were 8119 SNPs left (Table 2). These SNPs were filtered with a genotype quality (GQ) greater than 29, leaving 5297 SNPs (Table 2). A total of 5171 SNPs that had the contig sequences available were then prepared into shorter sequences with the SNP sites as the start positions for BLAST against the Tifrunner reference (ver. 1). Transcriptome sequences were cDNA sequences, which consisted of only the DNA sequences of the exons. After running BLAST against the genome reference, some results showed up as fragmented matches because the gaps were introns, making it challenging to trace the same SNP positions. Therefore, using the SNP site as the start position for BLAST was important to keep track of the target position. The purpose of running this first BLAST was to obtain the corresponding information about the chromosome and position on the Tifrunner reference (ver. 1) for each SNP.

After BLAST, sequences that had no match to the Tifrunner reference were removed, and the remaining 4802 sequences were then checked at their first positions to remove the ones that did not have a match at the SNP sites (Table 2). There were 3092 sequences left, and they were checked for the number of chromosomes mapped to (Table 2). Ideally, each sequence should map to at least two chromosomes, which are the target chromosome and its homoeologous chromosome, with zero to a few matches at the paralogous sites. At this step, sequences that were mapped to only one chromosome were removed, resulting in 514 sequences left (Table 2). This was because the SNPs used here were called in the past without examining whether they were true SNPs or not; therefore, obtaining base information from both the target site and its possible homoeologous site was important. The expected polymorphism should be among different accessions instead of between the target site and its homoeologous site. If the base on the target site was different from the base on the homoeologous site, there was a higher chance that this SNP was not a true SNP. For a better resolution of selecting true SNPs, it was important to compare the bases on both the target site and its possible homoeologous site and make sure they were the same. This might remove some true SNPs that had different bases on the target site and its homoeologous site, however. This step was not performed in the SNP chip dataset, because the SNP chip Affymetrix Axiom_Arachis2 was already designed to be able to distinguish the homoeologs [10]. The transcriptome SNP data were not examined in the past by any filtering methods such as SWEEP or BLAST to distinguish A and B genomes; therefore, there was a need to examine the SNP sites on both the target chromosome and its homoeologous chromosome to have higher confidence of selecting true SNPs. By completing this step, 279 sequences had BLAST results matched on both the target site and its possible homoeologous site (Table 2).

The information about the chromosome and position of each of the 279 SNPs was used for extracting flanking sequences using the Tifrunner reference (ver. 1). Each extracted sequence was 601 bases long, with 300 bases flanking each side of the SNP site. These 279 sequences were then used for the second BLAST run against the Tifrunner reference (ver. 1) with an e-value of 1 × 10^−50^ for a better search of paralogs. Running the second BLAST using Tifrunner sequences could provide a better search of the sequences on the homoeologous and paralogous regions. To be able to distinguish the targets from the homoeologous and paralogous reads, each sequence flanking the SNP site should be different from any of its homoeologous and/or paralogous sequences. Each of the remaining 180 sequences was then examined at the SNP site for bases on both A and B homoeologous positions (Table 2). To reduce the chance of selecting targets that are not true SNPs, a total of 160 SNPs that had the same bases on the homoeologous positions were kept based on the SNP data of the Tifrunner reference output from the BLAST results (Table 2). These 160 SNPs were selected as targets for designing probes and were named with a beginning of “T_”.

#### 2.1.3. KASP Data and WGS Data

There were five KASP markers used in this research, including three root-knot nematode (RKN [23]) resistance SNP markers and two fatty acid desaturase SNP markers (FAD2A and FAD2B [24]). The chromosome and position information about these five SNP targets was collected for designing probes and named with a beginning of “K_”. Also, 611 SNP targets from WGS using the ICRISAT reference data with Tifrunner (ver. 2) were included in this research [25]. These 611 SNPs were selected as targets for designing probes and were named with a beginning of “R_”. The chromosome and position information about these SNP targets from various sources was then combined. A total of 2904 targets were selected, and the information along with the references were sent to Tecan Allegro for the designing of custom probes (Table 3).

### 2.2. DNA Extraction

The Tecan Allegro Targeted Resequencing V2 kit is designed to include 48 samples at a time to process for one DNA library. To test the kit and run a trial experiment, 48 out of 192 reactions of the kit were used in this research. A total of 48 different peanut accessions were chosen for this trial experiment as representatives of different types of populations to check if there will be SNPs identified among closely related accessions, such as sister lines in a breeding population and some U.S. peanut minicore collections that shared similar admixture proportions based on their STRUCTURE graph (Sung and Burow, unpublished). These 48 genotypes were categorized into 16 groups, and DNA was prepared from seed samples including eight wild species accessions (seven diploid genotypes and one synthetic tetraploid genotype, TxAG-6) and 40 cultivated genotypes (Table 4).

DNA was extracted using a modified Qiagen (Qiagen Inc., Valencia, CA, USA) DNAeasy protocol for peanut seeds. The end of each seed was cut as approximately a 20 mg cotyledon piece from the distal end and placed into a 1.5 mL centrifuge tube. After adding 600 μL of peanut nuclei lysis buffer (PNLB) [42] into each tube, samples were ground with a pestle until no large pieces remained. Then 6 μL of RNase A (10 µg mL^−1^) was added, followed by vortexing, and then tubes were incubated for 10 min at 65 °C. Tubes were inverted two to three times during incubation. Then 200 μL of 5M KOAc pH 4.8 were added into each tube, followed by mixing and incubation for five minutes on ice. After incubation, the lysate was placed into a microcentrifuge and run for five minutes at 20,800× *g*. Then 400 μL of supernatant were transferred to a new 1.5 mL centrifuge tube without disturbing the cell debris pellet. After adding 600 μL of binding buffer AP3/E (one part 5M guanidine hydrochloride to which were added two parts ethanol before use), the sample was mixed immediately by pipetting, and 650 μL of the mixture were transferred to an EconoSpin DNA/RNA mini spin column (Epoch Life Science, Houston, TX, USA) placed in a collection tube. After centrifuging for one minute at 20,800× *g*, the flow-through was discarded. The remaining sample mixture was transferred to a spin column and centrifuged for one minute at 20,800× *g*. After discarding the flow-through, 500 μL of washing buffer AW/E (10mM Tris HCl pH 8.0, to which are added four parts ethanol) were added to wash away salts. After centrifuging for one minute at 20,800× *g*, the flow-through was discarded. Then 500 μL of Buffer AW/E were added again for a second wash, followed by centrifuging for two minutes at 20,800× *g*. Then the spin column was transferred to a new 1.5 mL centrifuge tube with 100 μL of elution buffer AE (10 mM Tris HCl pH 8.0) directly added onto the membrane. After five minutes of incubation at room temperature, each tube was centrifuged for one minute at 20,800× *g* to elute the DNA. The final tubes with 100 μL extracted DNA were stored in the −20 °C freezer.

The extracted DNA samples were measured for concentration using the QuantiFluor dsDNA system (Promega, Madison, WI, USA) on a Tecan F200 Infinite plate reader fluorometer (Tecan, Zürich, Switzerland).

### 2.3. Library Prep and Sequencing

#### 2.3.1. Enzymatic Fragmentation

The steps of the library preparation were completed by following the Tecan Allegro Targeted Genotyping V2 user guide (Publication Number: M01501; Revision: v2; https://www.tecan.com/doc/allegro-targeted-genotyping-v2-user-guide-pdf-m01501, accessed on 1 April 2022). Before starting, the 48 peanut genomic DNA samples were pipetted into a 96-well plate at a concentration of approximately 80 ng in 5 μL of nuclease-free water for each sample. The fragmentation master mix was made by combining 184.8 μL of fragmentation buffer mix and 79.2 μL of fragmentation enzyme mix. After adding 5 μL of fragmentation master mix to each DNA sample and mixing well, the 96-well plate was sealed and placed in a thermal cycler programmed to run Program 1 (Enzymatic Fragmentation: 25 °C—15 min, 75 °C—20 min, hold at 4 °C).

#### 2.3.2. Adapter Ligation

After Program 1, each of the 48 fragmented samples (10 μL) was mixed with a unique single-index barcoded adapter (3 μL). The ligation master mix was made by combining 158.4 μL ligation buffer mix, 39.6 μL ligation enzyme mix, and 118.8 μL nuclease-free water. After adding 6 μL ligation master mix to each sample and mixing well, the 96-well plate was sealed and placed in a thermal cycler programmed to run Program 2 (Adapter Ligation: 25 °C—30 min, 70 °C—10 min, hold at 4 °C).

#### 2.3.3. Probe Binding, Hybridization, and Extension

After Program 2, the 48 reactions were pooled into a single 2.0 mL Eppendorf LoBind tube with a total volume of approximately 900 μL and mixed with 450 μL of room-temperature Agencourt (Beckman-Coulter, Brea, CA, USA) beads. Beads carrying DNA were then washed with 70% ethanol. After the purification and elution steps, 32 μL of the purified pool were transferred to a 0.2 mL tube. The target extension master mix was made by combining 7 μL of target extension buffer mix and 10 μL of Allegro custom probe mix (number ST2291G_1). After adding 17 μL of target extension master mix to the 32 μL pooled sample and mixing well, the 0.2 mL tube was sealed and placed in a thermal cycler programmed to run Program 3 (Probe Binding, Hybridization, and Extension: 95 °C—5 min, 200 cycles (80 °C—10 s, decrease temp 0.1 °C each cycle), 60 °C hold, 72 °C—10 min, hold at 4 °C). It is important to note that the incubation step at 60 °C should be held for more than 12 h. While the tube was still in the thermal cycler at 60 °C, 1 μL target extension enzyme was added to the tube and mixed well before the thermal cycler was advanced to the 72 °C step.

#### 2.3.4. Library Amplification

After Program 3, post-enrichment purification steps were performed by adding 50 μL of room-temperature nuclease-free water to bring the total volume of the sample to 100 μL and mixing with 80 μL of room-temperature Agencourt beads. Beads were then washed by using 70% ethanol for purification. After the purification and elution steps, 24 μL of the enrichment pool was transferred into a fresh tube for further library amplification. To determine the appropriate number of library amplification cycles, real-time PCR was performed. The real-time PCR master mix was made by combining 13.2 μL amplification buffer mix, 5.28 μL amplification primer mix, 1.32 μL amplification enzyme mix, 2.64 μL amplification enhancer mix, 3.3 μL 20X EvaGreen (Biotium, CA, USA) reagent, and 27.06 μL nuclease-free water. The enrichment pool was diluted 1:8 by combining 2 μL of the enrichment pool with 14 μL of nuclease-free water. In this study, we made three duplicates of the diluted enrichment pools for real-time PCR. After adding 8 μL of the real-time PCR master mix to 2 μL of each of the three diluted enrichment pools and three no-template controls and mixing well, the qPCR plate was sealed and placed in the Roche LightCycler^®^ (F. Hoffmann-La Roche, Basel, Switzerland) 480 instrument programmed to run the real-time PCR program (37 °C—10 min, 95 °C—3 min, 35 cycles (95 °C—30 s, 62 °C—15 s, 72 °C—20 s)). The amplification plot was examined, and the cycle number for late exponential phase amplification was determined to be 22.

The appropriate number of library amplification cycles was 19, which was calculated by subtracting three cycles from the 22 cycles determined based on the real-time PCR result. The library amplification master mix was made by combining 20 μL amplification buffer mix, 8 μL amplification primer mix, 2 μL amplification enzyme mix, 4 μL amplification enhancer mix, and 46 μL nuclease-free water. After adding 80 μL of the amplification master mix to 20 μL of the enrichment pool and mixing well, the PCR tube was sealed and placed in a thermal cycler programmed to run Program 4 (Library Amplification: 37 °C—10 min, 95 °C—3 min, 19 cycles (95 °C—30 s, 62 °C—15 s, 72 °C—20 s), 72 °C—2 min, hold at 10 °C).

#### 2.3.5. Final Library Purification

After Program 4, the final library purification steps were performed by mixing the sample with room-temperature Agencourt beads for 70% ethanol purification. The eluted library was then checked for the quantitative and qualitative assessment by running 1 μL of 5 ng/μL of the library on a Tapestation 4200 platform with High Sensitivity D1000 ScreenTape (Agilent, Santa Clara, CA, USA) before sending it for sequencing. After confirming the library concentration, 20 μL of the sequencing-ready library (with an average size of 382 bp) with a concentration of 29.2 ng/μL and 10 μL of custom R1 primer initial stock at 100 μM were sent for MiSeq sequencing at the Texas A&M Veterinary Medicine Genomics Center.

### 2.4. Bioinformatics Data Analysis

All the bioinformatics analyses were completed using the laboratory Supermicro server, CentOS release 6.10, with 16 processor cores and 32GB RAM. A total of 96 fastq files were received, including two paired-end files for each of the 48 accessions, and the 23,035,880 raw reads were aligned against an artificial reference that consisted of 7755 contigs using the Burrows–Wheeler Aligner (BWA ver. 0.7.5a) [43]. The artificial reference was created using DNA sequences flanking the 2770 targets and their homoeologous and/or paralogous sites extracted from the Tifrunner references. Each contig sequence was 301 bases long, with 150 bases flanking each side of the target site. Paired-end alignments were created in the Sequence Alignment Map (SAM) format using BWA SAMPE, then the sam files were compressed in binary format to bam files using SAMtools (ver. 0.1.19) to save space [44]. After adding or replacing read groups, files were sorted to group reads that map to the same contig using SortSam in Picard Tools (ver. 1.98; http://broadinstitute.github.io/picard, accessed on 1 April 2022). Duplicate reads were marked and removed using MarkDuplicates, and the files were indexed using BuildBamIndex in Picard Tools, then the 48 bam files were processed to be ready for variant calling.

Variants were called using the Genome Analysis Toolkit (GATK ver. 2.8-1; https://gatk.broadinstitute.org/hc/en-us, accessed on 1 April 2022) Haplotype Caller to identify SNPs compared to the reference [45]. The 48 Variant Call Format (vcf) files were then merged into one single vcf file for identification of SNPs among genotypes. Data were then filtered by using two different criteria, hereafter called “Filter One” for the dataset with SNPs filtered out with a GQ less than 30 or a DP less than 10 and “Filter Two” for the dataset with SNPs filtered out with a GQ less than six or a DP less than two. Command lines in Bourne-Again Shell (bash) scripts and notes used for analysis are included on GitHub (https://github.com/JoyCJS/ReseqPipeline, accessed on 1 April 2022). Microsoft Excel (ver. 2206) and R (ver. 4.3.0) were used for further data analysis and visualization.

This approach employed sequence analysis steps that are also used in other GBS methods; therefore, these methods provide similar genotyping efficiency with built-up pipelines. Specifically, for peanut research, this approach helped remove highly similar genetic regions that could generate wrong SNP calls (not true SNPs), and we propose that this approach could maintain high genotyping accuracy by its feature to capture true SNPs.

## 3. Results

### 3.1. Custom Probe Design

Among the 2904 targets sent to Tecan Allegro, 5154 probes were designed successfully to cover 2770 targets, including 2093 targets from the U.S. minicore SNP chip data, 136 targets from the transcriptome RNA-Seq sequencing data, 536 targets from WGS, and five targets from KASP SNP data (Supplementary Table S1). The final design included 2384 targets covered by two probes and 386 targets covered by one probe. The targets covered by two probes had sequences read from both directions of the target SNP sites, while the targets covered by one probe were read from one of the two sides of the target SNP sites. Targets covered by two probes benefit from an increasing read depth for variant calling and can avoid artifacts from only reading in one direction. Two probes have redundancy in case one probe fails because the Tecan Allegro design was in silico only and was not verified experimentally to work prior to the sequencing experiment. These probes were received as a single tube of reagents included in the Tecan Allegro Targeted Resequencing V2 kit and were used during the steps of the library preparation for the trial experiment on 48 selected accessions.

### 3.2. Bioinformatics Data Analysis

**Figure 3:**
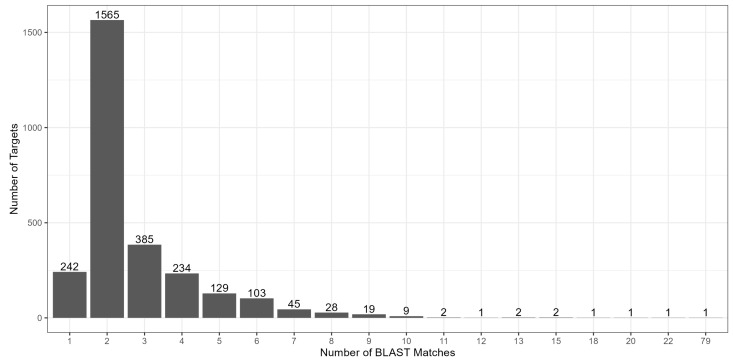
A bar plot showing that more than half of the targets had two BLAST matches.

**Figure 4:**
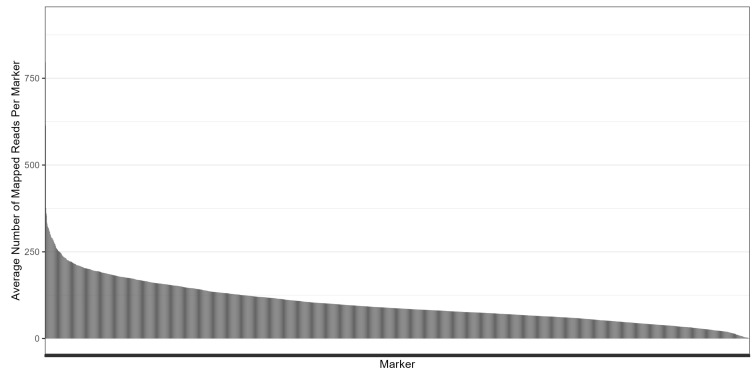
A bar plot showing the number of mapped reads averaged across 48 accessions for each marker (sorted from high to low).

On average, there were 2.8 matches per BLAST search across the 2770 target sequences, ranging from one to 79, with a median of two matches, making a total of 7755 sequences (Figure 3). Close to 90% of the targets had fewer than five BLAST matches. These 7755 sequences were then used as contigs (artificial chromosomes) and combined into an artificial reference, consisting of 2770 target contigs and 4985 homoeologous and paralogous contigs. Among the 23,035,880 raw reads, 13,402,453 reads (58.18%) were mapped to the artificial reference, with an average of 279,218 reads per accession and 4838 reads per target (including reads mapped to its homoeologous and paralogous contigs), resulting in an overall average of 36 reads per accession per contig. Among the 13,402,453 mapped reads, 8,825,651 reads (65.85%) were mapped to the target contigs with a range of two to 10,645 reads for each target, and the rest (4,576,802 reads; 34.15%) were mapped to the homoeologous and paralogous contigs. In general, the number of mapped reads averaged across 48 accessions for each marker ranged from 0.04 (M_727) to 911 (M_6224), with a mean of 101 and a median of 87 reads for each marker (Figure 4).

By using the 7755 sequences (301 bases long for each contig sequence, with 150 bases flanking each side of the targeted locus) as the artificial reference for variant calling, there were 1,332,912 SNPs called and merged into the final vcf file. These called SNPs were located on the 7755 contigs (artificial chromosomes) and were polymorphic between each accession and the reference (Tifrunner). Because the target sites were in the middle of each target sequence, SNPs at the target site were expected to be called at the 151^st^ positions on the artificial chromosomes made of the target sequences. Among these 2770 target sequences, 2638 of them had polymorphisms between the Tifrunner reference and at least one accession at the target sites (the 151^st^ positions), showing a recovery rate of 95%. After removing sites that had only missing data and the 0/0 genotype (reference genotype), there were 121,572 SNPs identified among 48 accessions located on 5372 contigs, including 2620 target sequences. Among these 2620 target sequences, 1547 of them had a SNP at the targeted locus (the 151^st^ position). These 1547 SNPs were used for further analyses due to the presence of polymorphism at the 151^st^ locus among the 48 accessions. The other 1073 sequences had no SNPs at the targeted locus but had additional SNPs in the flanking regions instead. The genotypic data of the 1547 SNPs were then compared within each of the 16 groups of peanut accessions for the identification of SNPs among closely related accessions within each group.

### 3.3. SNPs Identified Among Closely Related Genotypes

The overall numbers of SNPs identified from the 16 groups are listed in Table 5. In total, there were 1327 SNPs identified among 48 accessions using Filter One, and 1507 SNPs when Filter Two was used. Groups two to six included accessions (mostly minicore accessions) that showed similar admixture proportions in the STRUCTURE analyses of our SNP chip GWAS study (Sung and Burow, unpublished). Generally, there were fewer than 80 SNPs identified within each group, and all these SNPs were from the SNP chip and WGS datasets. There were two accessions in groups two to five, with a mean of 32.5 SNPs across groups when using Filter One and a mean of 50 SNPs across groups when using Filter Two. More SNPs were identified in group six than in groups two to five, which was expected because there was one more accession included in group six.

Groups seven to 10 included closely related sister lines for each of the four major U.S. market types. There were three accessions in groups seven to nine, with a mean of 51.7 SNPs across groups when using Filter One and a mean of 75.7 SNPs across groups when using Filter Two. As expected, there were fewer SNPs identified in group 10 than in groups seven to nine because there were only two accessions included in group 10. All these groups provided great estimations for genotyping in breeding programs. For example, TxL090206-41 from group nine was one of the accessions that performed well under water deficit stress [46], and TxL054520-27 from group 10 was used as a parent of one drought population, and it was also one of the accessions that performed well under water deficit stress [46]. These groups provided useful pilot insights demonstrating the use of this genotyping by resequencing platform for the MAS of peanut water deficit stress tolerance accessions.

Groups 11 to 13 included varieties commonly used as parents in breeding programs, and the results provided an estimation of useful SNPs and genetic variation between the varieties using this resequencing platform along with custom probes. Group 11 included three randomly picked seed samples of Tamspan 90, a bulked composite of 38 component lines. Based on the results, two of them were almost identical to each other (TS90-1 and TS90-2), showing very similar genotypes, while the third one (TS90-3) showed different genotypes from the other two samples. Surprisingly, there was so much variation among the component lines of a variety that plants appeared to be very similar phenotypically, suggesting that there was more variation than expected for accessions that look highly similar. The results revealed the ability of this platform to provide fine-scale genotyping for the determination of genotype similarity and variation. Group 12 included a single-seed drought test breeding line, TxL100212-03-03, and one of its parents, ICGS 76, which is tolerant to water deficit stress but shows a lower yield compared to TxL100212-03-03. The results showed a similar estimation of SNP numbers as the other groups with two accessions. Group 13 included four varieties, each from one of the four major U.S. market types; two of them were high oleic (OLin and Tamrun OL07), and the other two were low oleic (Jupiter and NMValC). These accessions were used as check varieties for FAD2A and FAD2B markers in the transcriptome sequencing study [22]. It is to be noted that SNPs were identified among any two of the accessions in the group; therefore, few of the SNPs were associated with high oleic traits. Based on the prior knowledge of oleic content in these accessions, SNPs with identical genotypes between OLin and Tamrun OL07 that were different from Jupiter and NMValC could be associated with high oleic phenotypes. Specifically, K_4 (FAD2A), included in this study as a control marker, was identified in group 13 (Filter Two), where the genotypes of OLin, Tamrun OL07, and NMValC were detected as A/A, A/A, and G/G, just as expected (G is the wild-type allele and A is the mutant-type allele; Supplementary Table S2). It was missing data for the K_4 genotype in Jupiter, likely due to its low coverage of mapped reads that was due to a low DNA concentration.

Group one included the two diploid accessions, *A. duranensis* (collection number 30067; A genome) and *A. ipaënsis* (collection number 30076; B genome), representing the parental species of the cultivated peanut, *A. hypogaea* (AB genome). The results of group one showed that targets on the A chromosomes were mostly matched to *A. duranensis*, while targets on the B chromosomes were mostly matched to *A. ipaënsis*. It was also noticed that, among all SNPs identified in group one, only one SNP target was located on the A genome chromosome, and all others were from the B genome chromosomes. Including more SNP markers by selecting new targets using the diploid references could help provide a better inference. The small numbers of SNPs between *A. duranensis* and *A. ipaënsis* may be explained by the fact that SNP targets in this study were designed to eliminate homoeologs between A and B genomes because they were not considered as true SNPs.

Group 15 included two samples of *A. magna* (B genome) that had different flower colors, K30097of (orange flowers) and K30097yf (yellow flowers). Both group one and group 15 included diploid accessions and showed few SNPs identified, which could be due to the low number of reads mapped to the reference. The possible reason that there were fewer reads could be because we used Tifrunner for target selection and mapping instead of using diploids references. Results also showed that the A genome accessions had most of the missing data from the SNPs that were selected from the Tifrunner B chromosomes, while both B and K genome accessions had most of the missing data from the SNPs that were selected from the Tifrunner A genome chromosomes, suggesting that the K genome had higher similarity to the B genome than the A genome.

Group 14 included five parental accessions (*A. cardenasii* GKP10017 (A genome), *A. diogoi* GK10602 (A genome), *A. batizocoi* K9484 (K genome), TxAG-6, and UF439-16-10-3-2), four offspring from the BC_1_ population (BC1-43, BC1-46, BC1-50, and BC1-60), and three offspring from the BC_3_ population (43-09-03-02, 60-02-03-02, and 63-04-02-02). TxAG-6 is a synthetic tetraploid accession derived from the cross [*A. batizocoi* × (*A. cardenasii* × *A. diogoi*)]^4×^ [39]. The three diploid parents are known to be root-knot nematode (RKN) resistant, whereas TxAG-6 inherited resistant alleles from them. The cultivated tetraploid parent, UF439-16-10-3-2, was a component line of Florunner, and was susceptible to the RKN. To introduce the resistant alleles to cultivated tetraploid peanuts, a backcross population was generated between TxAG-6 (donor parent) and UF439-16-10-3-2 (recurrent parent), and succeeding generations of the BC_1_ population were used to develop RKN-resistant cultivars such as COAN [47], NemaTAM [48], and Webb [49]. Similar to groups one and 15, there were diploid accessions, and therefore, a relatively lower number of SNPs were identified. This matched the heatmap in Supplementary Figure S1 (Supplementary Table S3), where diploid accessions had fewer reads mapped to the reference. Additionally, the results showed that there were more heterozygous genotypes in BC_1_ accessions than in BC_3_, which were more than in the parental accessions (Supplementary Table S2). This observation met biological assumptions and indicated good genotyping results for both homozygous and heterozygous genotypes using this platform. Furthermore, we identified all three RKN KASP markers (K_1, K_2, and K_3) that were included as control checks using the Filter Two criteria (Supplementary Table S2), suggesting that this platform was effective for genotyping in breeding populations.

Group 16 included only one variety, Tifrunner, the cultivated peanut reference genotype. Results of Tifrunner genotypes provided information about how accurate this genotyping system was by using the targeted resequencing platform. Results under Filter One showed that among the 1547 SNP targets, 1158 were missing data, 62 were data that did not pass the GQ or DP filtering, and 327 (21.1%) had genotype data. Among those 327 SNPs, 311 (95.1%) of them had a homozygous genotype (0/0) that matched the Tifrunner reference data, 15 had a heterozygous genotype (0/1), and one had a homozygous genotype (1/1) that did not match the Tifrunner reference data. Results under Filter Two showed that, among the 1547 SNP targets, 1158 were missing data, 16 were data that did not pass the GQ or DP filtering, and 373 (24.1%) had genotype data. Among those 373 SNPs, 354 (94.9%) of them had a homozygous genotype (0/0) that matched the Tifrunner reference data, 17 had a heterozygous genotype (0/1), and two had a homozygous genotype (1/1) that did not match the Tifrunner reference data. Based on the results, using Filter Two reduced the accuracy slightly from 95.1% to 94.9%, but most of the genotypes that had lower GQ or DP were still called correctly. Among all 1,332,912 SNPs called and merged into the final vcf file for Tifrunner, there were 922,615 SNPs that had missing data and 410,297 SNPs that had data regardless of the GQ or DP. Among the 410,297 SNPs, there were 405,748 SNPs showing the genotype 0/0, which was the reference genotype, and that indicated that around 98.9% of the SNPs were called to match the Tifrunner reference. These implied that Filter One provided higher quality results with strict thresholds, while Filter Two could still be accurate and useful for data analysis even if some SNPs had lower quality and/or read depth.

## 4. Discussion

Generally, the genotyping completed by the resequencing platform using the Tecan Allegro Targeted Resequencing V2 kit was able to identify polymorphic loci within each of the 16 groups of closely related genotypes. Additionally, most of the check targets (known KASP markers) could be identified as expected, indicating that this platform is a reliable tool for genotyping. The results of Tifrunner were helpful for examining how accurate the SNPs were called. Although there were many missing data, most of the genotyping results were correct, and the accuracy was high. However, the results of many groups reflected that missing data could be an issue in this study, even if there were a decent number of reads mapped. Considering that each sequence read was 151 bases long, while each contig was 301 bases long, there could be two scenarios for each contig mapping. If reads were mostly mapped to cover the same region, the other regions on the contig would have only a few reads to no coverage. On the other hand, if reads were mapped to different regions that cover the entire contig, the read depth on the target site could be reduced. Furthermore, an optional tool argument in the GATK HaplotypeCaller program—standard-min-confidence-threshold-for-calling (-stand-call-conf)—was set at 20 (the default was 30) and used in this study. This filtered out variant sites with a QUAL (the phred-scaled SNP/indel call quality) of less than 20, and only those with greater quality would be called. The above are possible explanations for the missing data in this study. Setting a lower phred-scaled confidence threshold for a higher sensitivity of SNP calling might help obtain more SNPs, and sequencing for a greater number of reads would also help by increasing the overall coverage.

The overall mapping rate in this study is 58.18%, which is acceptable, but it could be higher if we mapped the reads against the whole Tifrunner reference genome instead of the selected 7755 contigs. But it would increase the number of reads mapped to paralogs. Genomic differences between accessions and Tifrunner could also result in more unmapped reads, especially diploid accessions and their related backcross offspring, suggesting that this platform works better for tetraploids. Most of the mapped reads were aligned to their corresponding top two BLAST sequences (assumably, targets and their homoeologous sequences), indicating that this platform can catch most of the highly similar reads and distinguish true SNPs.

In general, a less-stringent e-value (such as 1 × 10^−10^ or 1 × 10^−2^) could be used for identifying paralogs with more BLAST hits; however, based on the number of BLAST matches observed in this study, using 1 × 10^−50^ as the threshold identified a decent number of paralogs. The results showed that most of the mapped reads were aligned to the top two or three significant BLAST matches, suggesting that identifying more paralogs with a larger e-value might not be necessary. Additionally, M_8171, which matched 79 BLAST hits (including one target, one homoeolog, and 77 paralogs), was included and used as a random check marker in this study, and the results revealed that all the 356 reads were mapped to the target sequence. This implied that using 1 × 10^−50^ as the threshold can identify enough paralogous regions for peanut accessions, while ensuring high-quality significant BLAST hits. In addition, the customized probes used in this study were demonstrated to successfully distinguish and avoid paralogous regions that are highly similar to the target sequences, indicating that this is a reliable genotyping platform.

It is to be noted that part of our BLAST steps should be modified for a better target selection in the transcriptome dataset. There are two BLAST searches required in our pipeline for target selection in the transcriptome dataset, and the purpose of the first BLAST is to obtain information about the chromosome number and position on the Tifrunner reference from the best hit for each locus. In this study, we used OLin cDNA sequences to run the first BLAST against the Tifrunner reference, and 2578 sequences (83%) had only one BLAST hit with a relatively lower score (higher e-value) due to gaps of the intron regions. We removed these sequences after the first BLAST because, preferably, we want at least two matches (with one on the A chromosome and another on the corresponding B chromosome) to compare the homoeologous sequences for better filtration of true SNPs. However, this step should be completed after the second BLAST, which uses the sequences extracted from the Tifrunner genome against the reference itself to avoid removing targets due to gaps in intron regions. With those gaps, even the best BLAST hits could have low scores; therefore, we should extract the sequences using the information from the best BLAST hits and run the second BLAST before removing sequences that had only one BLAST match.

While we have demonstrated that this platform is suitable for genotyping closely related offspring lines in a population, it is also important to know whether these targets detect enough polymorphisms between selected parents for a breeding program. In this study, COC080 and COC082 were chosen to be included in group three because they had similar admixture proportions, and there were eight SNPs identified (when using Filter One and 28 when using Filter Two) between them. However, there were 99 SNPs identified (when using Filter One; 122 when using Filter Two) between COC080 and COC760, and 82 SNPs identified (when using Filter One and 116 when using Filter Two) between COC080 and Schubert, indicating that more polymorphisms can be detected between two accessions that have more variation. Furthermore, we identified 85 SNPs (when using Filter One and 114 when using Filter Two) between two Spanish varieties, Schubert and OLin, while there were only 20 SNPs identified (when using Filter One and 40 when using Filter Two) between Schubert and its sister line in group 10. However, we noticed that around half of the loci were missing data between the above sets. Assuming that the genotype data were complete with less missing data, we predicted that there would be around 200 SNPs or more for each of the above sets, which could be more applicable for a QTL mapping. This could suggest the need for a greater read depth. MiSeq was used in this study, and for more accessions, using HiSeq, NextSeq, or NovaSeq may provide more read depth.

In this study, 8,825,651 reads were mapped to the 2770 target contigs, making the average coverage 66.38 reads per accession per target contig. This seems convincing; however, whether these reads provide enough SNPs depends on the population size and the variation among individuals. Based on the datasets in this pilot study (with MiSeq v2 PE 2 × 150 platform), reads that were 300 bases long might help increase the mapping accuracy and target site coverage. We suggest obtaining more reads to ensure enough coverage at target sites for SNP identification using a sequencing platform such as NextSeq 2000 SE 1 × 300 for a population of 192 samples. This provides around 100 million reads with a read length of 300 bases and can bring the average coverage to approximately 72 to 100 reads per accession per target contig or more. Furthermore, it would cost around USD 4562 in total for 192 samples, including the Tecan Allegro Targeted Resequencing V2 kit (USD 2264) and sequencing cost (USD 2298), resulting in USD 23.76 per sample. Using a larger population could be even more economical with a lower cost per sample. Additionally, if only targets with known polymorphisms between specific parents are used, the cost could be further lowered. This suggests that there were around 1500 targets with SNPs detected to replace those that were not polymorphic, and this might reduce the sequencing cost to around USD 18 per sample if calculating for 384 samples using a NextSeq 200 million reads platform.

This pilot study provides an overview of the availability to use this platform for peanut breeding programs with high genotyping accuracy and a decent recovery rate of selected targets. For closely related accessions, the SNPs identified can be used as variety-specific check markers for examining the purity of the seeds collected for an accession and distinguishing possible contamination to help maintain the homogeneity of variety seeds. Additionally, the custom probes provide flexibility to add or drop any marker, which means the same or different sets of markers can be assigned for each sequencing run. Comparing the SNP chip array, which only detects a fixed set of markers, and the RAD-Seq approach, which identifies different markers each run, this targeted resequencing seems to be a suitable system for genotyping to become a routine tool for peanut breeding programs on the basis of a cost-effective and high-throughput approach (Table 6). With the sequences captured by probes designed to recognize useful target SNPs, it reduces the need to pay for sequences that are flanking regions of no interest, which further reduces the cost compared to using a higher number of reads or loci that are already known to be not useful. The major drawback of this approach is that it requires pre-existing SNP data for target selection and the design of custom probes. To expand this study further, we plan to run 144 samples using the rest of the kit for a population, and we also plan to select more targets to be included in future designs. One important comparison that should be included in the future is to run the exact same DNA in two or three duplicates to look for the number of SNPs detected, and then it should be used as a baseline threshold number of possible background noise. This would give a good estimate of which targets could detect polymorphisms for the population and thus be beneficial for future larger populations and more breeding programs with a lower cost.

## 5. Conclusions

The development of a set of useful molecular markers for targeted traits can reduce expenses and save effort compared with larger data sets. There are many SNPs that distinguish peanut tetraploid accessions and diploid wild species. Some species in the section *Arachis* are more tolerant to water deficit stress, such as *A. duranensis* and *A. ipaënsis*, and it would be useful to include some diploid genotypes for target selection in the future [6,50,51]. These SNPs can be further designed into KASP markers for MAS in peanut breeding programs such as for water deficit stress tolerance breeding. This trial experiment only used 48 genotypes out of the 192 reactions; the remaining 144 reactions will be tested afterward, and more SNPs will be identified. The utility of the SNPs can be further tested by constructing linkage maps and QTL maps for breeding populations. The cost-effective genotyping platform in this research could make genotyping a more affordable choice for peanut breeding research.

## Figures and Tables

**Table 1:** The number of remaining target sites for each filtering step using SNP chip data.

Minicore SNP Chip Data Filtering Steps	# Sites
Total raw sites	47,837
Only select “PolyHighResolution”	8189
BLAST matches < 56 (each query)	5653
PIC > 30% for the SNP target	3339
Matched to Tifrunner with the target site	3339
With distinguishable paralogs/homoeologs	3178
True SNPs (final targets)	2128

**Table 2:** The number of remaining SNPs for each filtering step using transcriptome data.

Transcriptome SNP Data Filtering Steps	# SNPs
Total raw SNPs	306,820
Polymorphic among 12 tetraploid accessions	87,782
PIC > 30%	73,872
Overall DP > 99	43,946
Individual DP > 10	8119
Individual GQ > 30	5297
With available contig sequences	5171
BLAST matched to Tifrunner	4802
Matched to Tifrunner with the SNP site	3092
Mapped to more than one chromosome	514
Matched target/homoeologous chromosomes	279
With distinguishable paralogs/homoeologs	180
True SNPs (Final targets)	160

**Table 3:** A total of 2904 targets were selected and sent to Tecan Allegro for the designing of custom probes.

SNPs Sources	Polymorphic in A Genome	Polymorphic in B Genome	Total
SNP Chip	872	1256	2128
Transcriptome	100	60	160
KASP SNPs	4	1	5
WGS	353	258	611
Total	1329	1575	2904

**Table 4:** The list of 48 genotypes categorized into 16 groups with DNA concentration in ng/μL.

Group	#	Genotypes	Notes	Descriptions	Conc.
1	1	*A. duranensis* GKBSPSc 30067	A genome	Diploid parents	16
	2	*A. ipaënsis* GKBSPSc 30076	B genome		16
2	3	Georgia-09B [26]	run ^ǂ^	Similar admixture proportions (runner)	16
	4	COC155B (PI502111) [27]	run		16
3	5	COC080 (PI494018) [28]	Spa	Similar admixture proportions (Spanish)	16
	6	COC082 (PI494034) [28]	Spa		16
4	7	COC233 (PI290536) [29]	run	Similar admixture proportions (Virginia/bunch)	16
	8	COC246 (PI343398) [28]	Vir bun (Spa bun)		16
5	9	COC038 (PI493581) [29]	Val	Similar admixture proportions (Valencia)	16
	10	COC053 (PI493729) [28]	Val		16
6	11	COC310 (PI337406) [28]	Val	Similar admixture proportions (Valencia)	16
	12	COC334 (PI159786) [28]	Val		16
	13	COC760 (PI471952) [28]	Val-Spa		16
7	14	TxL054529-27	Val	Valencia sister lines	16
	15	TxL054529-33	Val (TAMVal OL14 [30])		16
	16	TxL054529-48	Val		16
8	17	TxL080243-06	run (Tamrun OL19 [31])	runner sister lines	16
	18	TxL080287-05	run		6.30
	19	TxL080256-02	run (Tamrun OL18L [31])		16
9	20	TxL090106-15	Vir	Virginia sister lines	16
	21	TxL090105-07	Vir		16
	22	TxL090206-41	Vir		16
10	23	TxL054520-27	Spa	Spanish sister lines	16
	24	Schubert [32]	Spa		16
11	25	Tamspan 90 [33]	Spa (TS90-1)	Bulk variety of 38 lines	16
	26	Tamspan 90	Spa (TS90-2)		10.77
	27	Tamspan 90	Spa (TS90-3)		16
12	28	TxL100212-03-03	Single seed (Higher yield)	Drought test breeding line and the parent	16
	29	ICGS 76 [34]	Drought parent (Lower yield)		16
13	30	OLin [35]	High O/L Spa	High and low O/L checks for FAD2A and FAD2B; Four cultivars represented four market classes in the Southwestern United States	16
	31	Tamrun OL07 [36]	High O/L run		16
	32	Jupiter [37]	Low O/L Vir		5.13
	33	NMValC [38]	Low O/L Val		16
14	34	*A. cardenasii* GKP10017	RKN resistant	Parents of TxAG-6	16
	35	*A. diogoi* GK10602			16
	36	*A. batizocoi* K9484			16
	37	TxAG-6 [39]	RKN resistant		16
	38	UF439-16-10-3-2	RKN susceptible	Florunner component line recurrent parent	16
	39	BC1-43 [40]	BC_1_	BC_1_ lines from TxAG-6 × UF439-16-10-3-2 cross	16
	40	BC1-46	BC_1_		16
	41	BC1-50	BC_1_		16
	42	BC1-60	BC_1_		16
	43	BC3-43-09-03-02 [40]	BC_3_	BC_3_ lines from TxAG-6 × UF439-16-10-3-2 cross	16
	44	BC3-60-02-03-02	BC_3_		16
	45	BC3-63-04-02-02 [40]	BC_3_		16
15	46	*A. magna* K30097of	Orange flowers	Different flower colors	6.61
	47	*A. magna* K30097yf	Yellow flowers		16.25
16	48	Tifrunner [41]	run	Reference accession	16

^ǂ^ Vir—Virginia; run—runner; Val—Valencia; Spa—Spanish; O/L—oleic/linoleic seed fatty acid ratio.

**Table 5:** The overall number of SNPs identified in each of the 16 groups.

Group	Filter ^ǂ^	SNP Chip	RNA-Seq	KASP	WGS	Total
1	One	3	0	0	0	3
	Two	8	0	0	3	11
2	One	41	0	0	5	46
	Two	45	0	0	11	56
3	One	6	0	0	2	8
	Two	20	0	0	8	28
4	One	37	0	0	8	45
	Two	54	0	0	15	69
5	One	29	0	0	2	31
	Two	42	0	0	5	47
6	One	47	0	0	14	61
	Two	58	0	0	18	76
7	One	73	0	0	9	82
	Two	96	0	0	18	114
8	One	34	0	0	4	38
	Two	52	0	0	12	64
9	One	25	0	0	10	35
	Two	35	0	0	14	49
10	One	18	0	0	2	20
	Two	31	0	0	9	40
11	One	52	0	0	10	62
	Two	78	1	0	21	100
12	One	22	0	0	3	25
	Two	34	0	0	8	42
13	One	364	0	0	36	400
	Two	513	1	1	61	576
14	One	201	2	2	70	275
	Two	270	3	3	134	410
15	One	2	0	0	0	2
	Two	8	0	0	3	11
16	One	5	0	0	11	16
	Two	7	0	0	12	19

^ǂ^ Filter One: GQ ≥ 30 and DP ≥ 10; Filter Two: GQ ≥ 6 and DP ≥ 2.

**Table 6:** A comparison of major differences between GBS, SNP chip, and Tecan Allegro Targeted Resequencing.

	Genotyping by Sequencing	Affymetrix Array SNP Chip	Tecan Allegro Targeted Resequencing
Consistency—Use the same markers across runs?	No	Yes	Yes
Flexibility—Does it allow to add or drop markers?	No	No	Yes
In-house library prep?	Yes	No	Yes
Estimated cost per plant?	USD 30–53	USD 25–28	USD 18–23
Number of SNPs generated?	5K–50K or more	5K–50K or more	2K–10K depending on design

## Data Availability

Assemblies and variant information described in this manuscript have been deposited at Figshare at the following URL: https://dx.doi.org/10.6084/m9.figshare.26801122, accessed on 1 April 2022 (and will be released upon acceptance of the manuscript for publication). SAM files of raw read data for each of the accessions will be deposited in the NCBI SRA database under bioproject PRJNA605472. Command lines in bash scripts and notes used for the 48 samples analysis are included on GitHub (https://github.com/JoyCJS/ReseqPipeline, accessed on 1 April 2022).

## Supplementary Materials

The following supporting information can be downloaded at: https://www.mdpi.com/article/10.3390/genes15111364/s1. Table S1. Information of 5154 probes designed to cover 2770 targets. Table S2. Results of genotypes for each target of each accession. Table S3. Sorted matrix of the number of mapped reads. Figure S1. Heatmap showing the number of reads mapped to contigs for each accession, with bar plots showing the distribution of average number of mapped reads sorted from high to low.
