# Supplementary figures and images for: Reduced-Cost Genotyping by Resequencing in Peanut Breeding Programs Using Tecan Allegro Targeted Resequencing V2
Source: Genes (Basel). 2024 Oct 24;15(11):1364. doi: 10.3390/genes15111364 (PMC11593647; doi:10.3390/genes15111364)

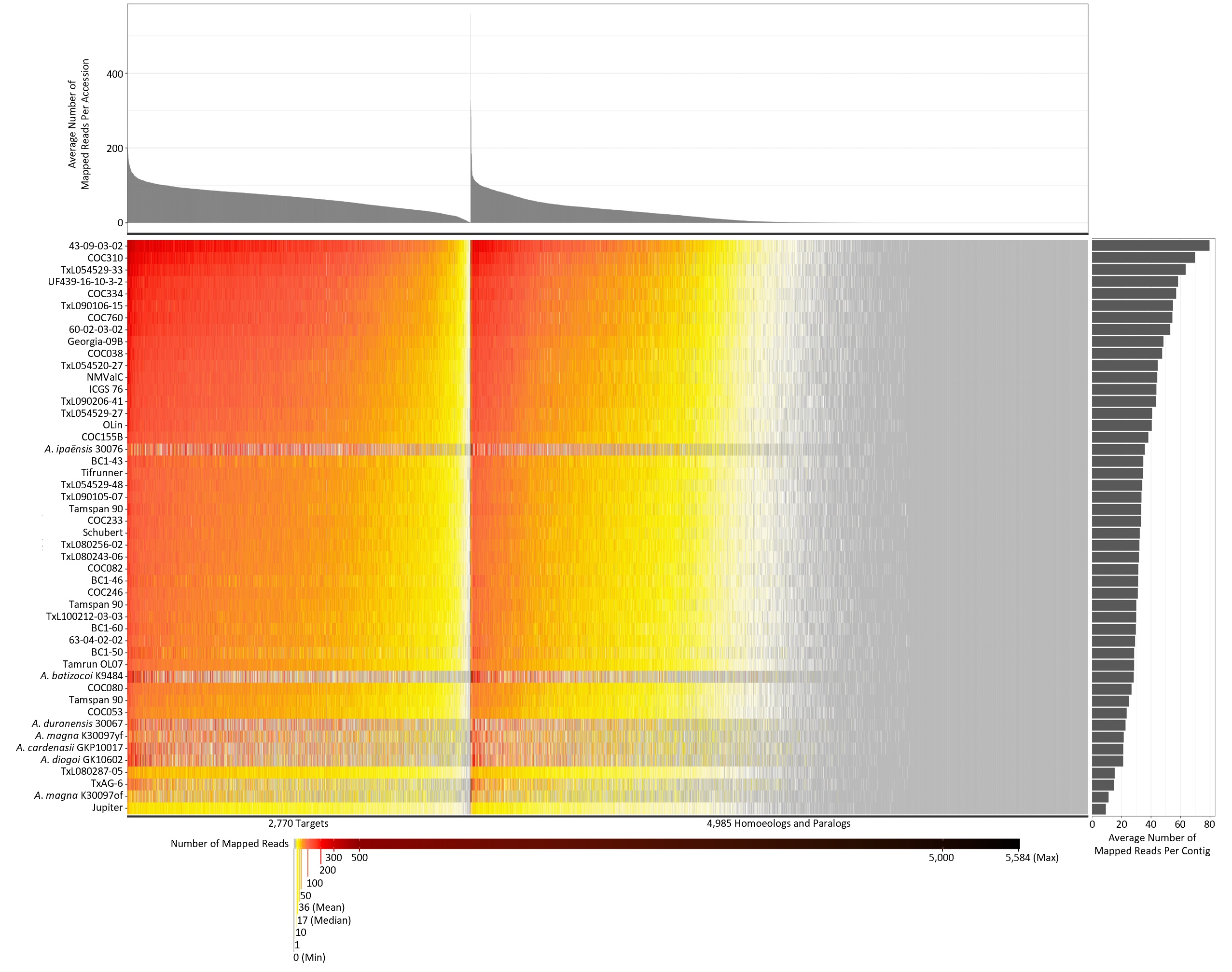

Supplement: Supplementary file 1 [file genes-15-01364-s001.zip › Figure_S1.jpg]
